# St. Vitus’ Dance

**Published:** 1883-09

**Authors:** 


### ST. VITUS DANCE.

ST. VITUS DANCE (Chorea) is characterized by an irregular contraction of the voluntary muscles. This contraction is at first confined to a few muscles on one side of the upper part of the body, perhaps even to those of the fingers. It gradually extends, until most of the voluntary muscles of the upper extremities, and less often those of the lower extremities and the trunk, are affected.

In rare cases, especially in adults, the movements are exceedingly and dangerously violent. In the less violent, they may impair speech, render locomotion and the necessary acts of life impossible. They may also produce the most ludicrous grimaces and the most grotesque gesticulations.

The appetite is apt to be poor and the temper irritable. Sometimes the mind is temporarily impaired. Though the disease may occur even in infancy and extreme old age, yet it is mainly confined within the ages of ten and fifteen. It is very much more frequent in girls than in boys.

Its cause is not certain. In most cases there has been found to be a deficiency in the red globules of the blood. Fright, a fit of anger, and other excitements have brought on an attack.

As to its duration, a medical writer says it "varies from a few weeks to several months. The average duration is from two to three months. In the vast majority of cases the termination is in recovery. In a very small proportion of cases it becomes chronic and is incurable. Relapses are apt to occur after intervals varying from a few months to two or three years. As a rule, the relapses are shorter than the primary attacks."

As to remedies, all known causes must first be removed; ample nutrition, daily exposure, and, if practicable, exercise in the open air, should be secured, and a physician should prescribe the medicine that should be used.—*Youths' Companion.*

